# Early extubation with immediate non-invasive ventilation versus standard weaning in intubated patients for coronavirus disease 2019: a retrospective multicenter study

**DOI:** 10.1038/s41598-021-92960-z

**Published:** 2021-06-28

**Authors:** Gianmaria Cammarota, Rosanna Vaschetto, Danila Azzolina, Nello De Vita, Carlo Olivieri, Chiara Ronco, Federico Longhini, Andrea Bruni, Davide Colombo, Claudio Pissaia, Federico Prato, Carlo Maestrone, Matteo Maestrone, Luigi Vetrugno, Tiziana Bove, Francesco Lemut, Elisa Taretto, Alessandro Locatelli, Nicoletta Barzaghi, Martina Cerrano, Enrico Ravera, Christian Zanza, Andrea Della Selva, Ilaria Blangetti, Francesco Salvo, Fabrizio Racca, Yaroslava Longhitano, Annalisa Boscolo, Ilaria Sguazzotti, Valeria Bonato, Francesca Grossi, Federico Crimaldi, Raffaella Perucca, Ester Boniolo, Federico Verdina, Gian Luca Vignazia, Erminio Santangelo, Riccardo Tarquini, Marta Zanoni, Antonio Messina, Matteo Marin, Paola Bacigalupo, Graziana Sileci, Nicolò Sella, Edardo De Robertis, Francesco Della Corte, Paolo Navalesi

**Affiliations:** 1 Dipartimento di Medicina e Chirurgia, Università degli Studi di Perugia, Perugia, Italy; 2 Department of Anesthesia and Intensive Care, AAzienda Ospedaliero Universitaria “Maggiore della Carità”, Novara, Italy; 3 Translational Medicine Department, Università “del Piemonte Orientale”, Novara, Italy; 4 Anesthesia and Intensive Care, Ospedale Sant’Andrea”, Vercelli, Italy; 5 Department of Medical and Surgical Science, Università “Magna Graecia”, Catanzaro, Italy; 6 Department of Anesthesia and Critical Care, Ospedale “Ss. Trinità”, Borgomanero, Italy; 7 Department of Anesthesia and Critical Care, Ospedale “degli Infermi”, Biella, Italy; 8 Department of Anesthesia and Critical Care, Presidio Ospedaliero Domodossola e Verbania “ASL VCO”, Domodossola-Verbania, Italy; 9 Department of Medicine, Anesthesia and Intensive Care Clinic, Università di Udine, Udine, Italy; 10 Department of Anesthesia and Critical Care, Ospedale “Monsignor Giovanni Galliano”, Acqui Terme, Italy; 11 Department of Anesthesia and Critical Care, Azienda Ospedaliera “Santa Croce e Carle”, Cuneo, Italy; 12 Department of Emergency Medicine-Anesthesia and Critical Care-Michele, Pietro Ferrero Hospital, Verduno, Italy; 13 Department of Anesthesia and Intensive Care, Azienda Ospedaliera SS. Antonio e Biagio e Cesare Arrigo, Alessandria, Italy; 14 Department of Anesthesia and Intensive Care, Ospedale “Regina Montis Regalis”, Mondovì, Italy; 15 Anesthesia and Intensive Care Unit, Ospedale Universitario di Padova, Padova, Italy; 16 Humanitas, Clinical and Research Center – IRCCS, Rozzano, Milan Italy; 17 Department of Medicine-DIMED, Università di Padova, Padova, Italy

**Keywords:** Respiratory distress syndrome, Viral infection

## Abstract

In patients intubated for hypoxemic acute respiratory failure (ARF) related to novel coronavirus disease (COVID-19), we retrospectively compared two weaning strategies, early extubation with immediate non-invasive ventilation (NIV) versus standard weaning encompassing spontaneous breathing trial (SBT), with respect to IMV duration (primary endpoint), extubation failures and reintubations, rate of tracheostomy, intensive care unit (ICU) length of stay and mortality (additional endpoints). All COVID-19 adult patients, intubated for hypoxemic ARF and subsequently extubated, were enrolled. Patients were included in two groups, early extubation followed by immediate NIV application, and conventionally weaning after passing SBT. 121 patients were enrolled and analyzed, 66 early extubated and 55 conventionally weaned after passing an SBT. IMV duration was 9 [6–11] days in early extubated patients versus 11 [6–15] days in standard weaning group (*p* = 0.034). Extubation failures [12 (18.2%) vs. 25 (45.5%), *p* = 0.002] and reintubations [12 (18.2%) vs. 22 (40.0%) *p* = 0.009] were fewer in early extubation compared to the standard weaning groups, respectively. Rate of tracheostomy, ICU mortality, and ICU length of stay were no different between groups. Compared to standard weaning, early extubation followed by immediate NIV shortened IMV duration and reduced the rate of extubation failure and reintubation.

## Introduction

The rapid pandemic spread of coronavirus disease 2019 (COVID-19) caused by severe acute respiratory syndrome coronavirus 2 (SARS-CoV-2) represents a global public health emergency. A large number of COVID-19 patients require hospitalization for hypoxemic acute respiratory failure (ARF)^1–3^, with about 15% of cases needing invasive mechanical ventilation (IMV) in intensive care unit (ICU)^3^. While the best strategy of mechanical ventilation is still debated^4–6^, data on how to wean COVID-19 patients from IMV are even more scarce.

Usually, weaning off IMV starts with a spontaneous breathing trial (SBT) to test whether the patient is able to maintain spontaneous unassisted breathing^7^. Non-invasive ventilation (NIV) has been proposed as a valid tool to reduce the time spent on invasive ventilation in patients recovering from hypercapnic ARF^8,9^. More recently, early extubation followed by immediate NIV, in patients still dependent on relatively high level of positive end-expiratory pressure and inspiratory assistance, has been safely and effectively used in selected patients recovering from hypoxemic ARF, with a reduction of IMV duration and hospital length of stay, and a decrease of pulmonary infections (ventilator associated pneumonia or tracheobronchitis), compared to standard weaning through the endotracheal tube^10,11^.

In principle, early extubation followed by immediate NIV might be useful in intubated COVID-19 patients with ARF. In this retrospective multicenter observational study, we therefore compared early extubation followed by immediate NIV and standard weaning with respect to duration of IMV (primary endpoint), rate of extubation failure, reintubation, and tracheostomy, intensive care unit (ICU) mortality, and length of stay (additional endpoints).

## Results

**Figure 1:**
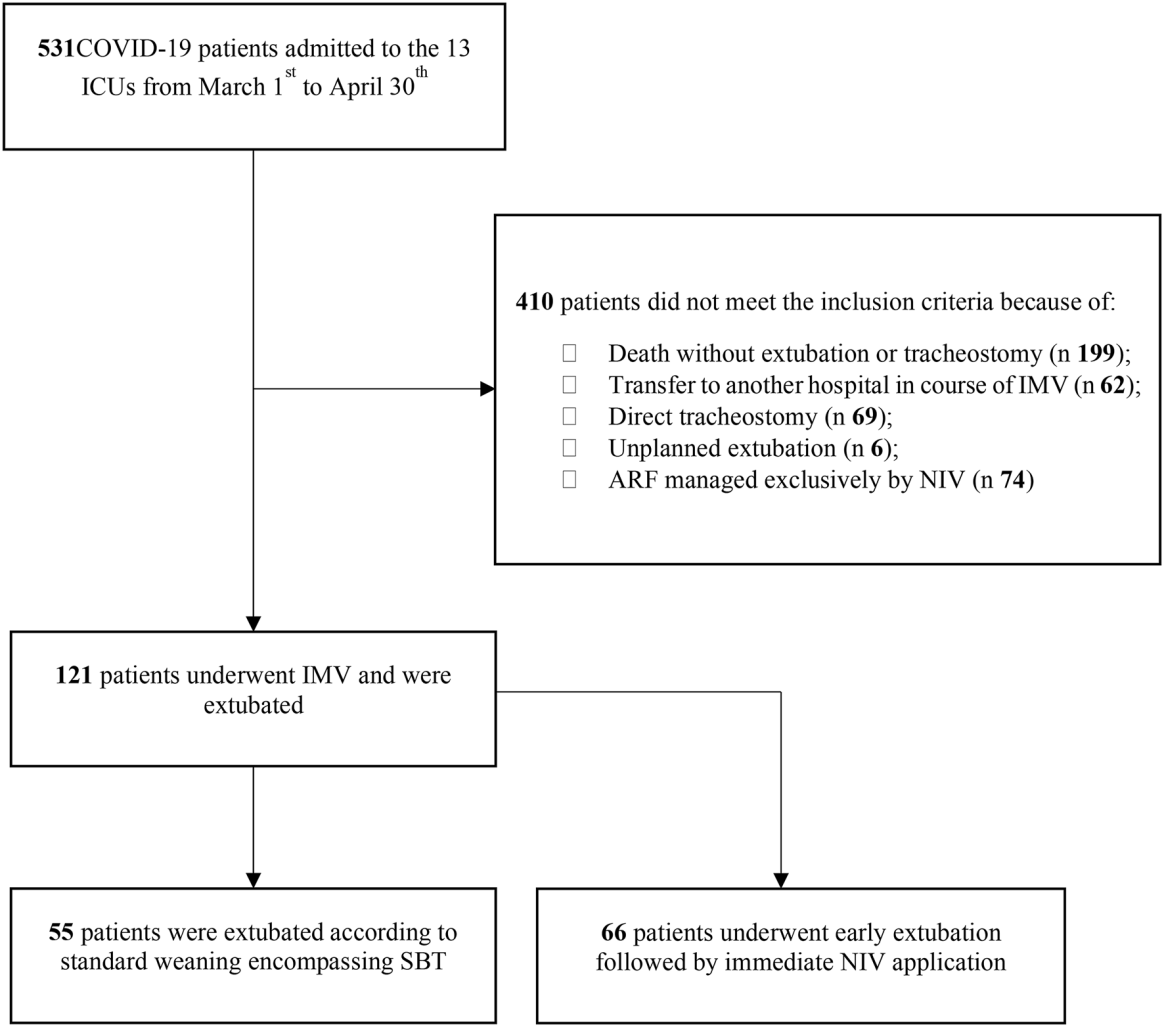
Study flow chart. COVID-19, coronavirus disease 2019; ICU, intensive care unit; ARF, acute respiratory failure; NIV, non-invasive ventilation; IMV, invasive mechanical ventilation; SBT, spontaneous breathing trial.

COVID-19, coronavirus disease 2019; ICU, intensive care unit; ARF, acute respiratory failure; NIV, non-invasive ventilation; IMV, invasive mechanical ventilation; SBT, spontaneous breathing trial.

From March 1st to April 30th, 2020, 531 patients were admitted to the participant ICUs, of whom 121 were enrolled and finally analyzed (Fig. 1).

Among these, 55 patients were extubated according to standard weaning strategy after encompassing SBT, whereas 66 patients underwent early extubation and immediate NIV. Demographic and baseline clinical characteristics are reported in Table 1. In the standard weaning group, a higher incidence of active smoking (*p* < 0.001), chronic arterial hypertension (*p* = 0.001), ischemic heart disease (*p* = 0.008), and other medical disease (*p* = 0.045) were reported, compared to the early extubation group. Also, the length of NIV before intubation was longer in the standard weaning group (*p* = 0.003).

**Table 1:** Demographic and baseline clinical characteristics.

	Standard weaning (n = 55)	Early extubation (n = 66)	*P* value
**Characteristics**			
Age, years	64.0 [58.0–69.0]	62.5 [54.8–70.3]	0.514
Male, n (%)	49 (89.1)	48 (72.7)	0.038
BMI, kg*m^−2^	27.8 [24.0–30.1]	26.6 [24.3–30.5]	0.650
SAPS II	40.0 [30.0–46.0]	34.0 [29.0–42.0]	0.212
SOFA	7.0 [3.5–8.0]	5.0 [3.0–8.0]	0.550
Comorbidities			
Chronic arterial hypertension, n (%)	36 (65.5)	23 (34.9)	0.001
Ischaemic heart disease, n (%)	6 (10.9)	0 (0.0)	0.008
Diabetes, n (%)	10 (18.2)	11 (16.7)	> 0.999
COPD, n (%)	7 (12.7)	2 (3.0)	0.077
Kidney disease, n (%)	2 (3.6)	1 (1.5)	0.590
Obesity (BMI ≥ 30 kg*m^−2^), n (%)	18 (32.7)	17 (25.8)	0.427
Other medical disease, n (%)	33 (60.0)	27 (40.9)	0.045
Active smoke, n (%)	22 (40.0)	8 (12.1)	< 0.001
Time lag between symptoms onset and hospital admission, days	6.4 (5.0–9.5)	7.0 (4.0–10.0)	0.918
NIV duration before intubation, days	2.0 (1.0–3.0)	1.0 (0.0–2.3)	0.003

Data are presented as number and percentage of patients (in brackets) or median and interquartile range [in brackets]. BMI, body mass index; SAPSII, simplified acute physiology score II, SOFA, sequential organ failure assessment; COPD, chronic obstructive pulmonary disease; NIV, non–invasive ventilation. *P* values refer to Fisher’s exact test or Mann–Whitney test.

Clinical outcomes are presented in Table 2. In the standard weaning group, the length of IMV was longer (*p* = 0.034) and the extubation failure (*p* =  0.002) and reintubation rate (*p* = 0.009) were higher compared to the early weaning group.

**Table 2:** Clinical outcomes.

	Standard weaning (n = 55)	Early extubation (n = 66)	*P* value
Invasive mechanical ventilation duration, days	11.0 [6.0–15.0]	9.0 [6.0–11.0]	0.034
Extubation failure, n (%)	25 (45.5)	12 (18.2)	0.002
Reintubation, n (%)	22 (40.0)	12 (18.2)	0.009
Tracheostomy, n (%)	13 (23.6)	7 (10.6)	0.084
ICU length of stay, days	15.0 [9.0–21.0]	13.5 [9.8–20.0]	0.514
ICU mortality, n (%)	6 (10.9)	6 (9.1)	0.769

Data are presented as number and percentage of patients (in brackets) or median and interquartile range [in brackets]. ICU, intensive care unit. *P* values refer to Fisher’s exact test or Mann–Whitney test.

**Figure 2:**
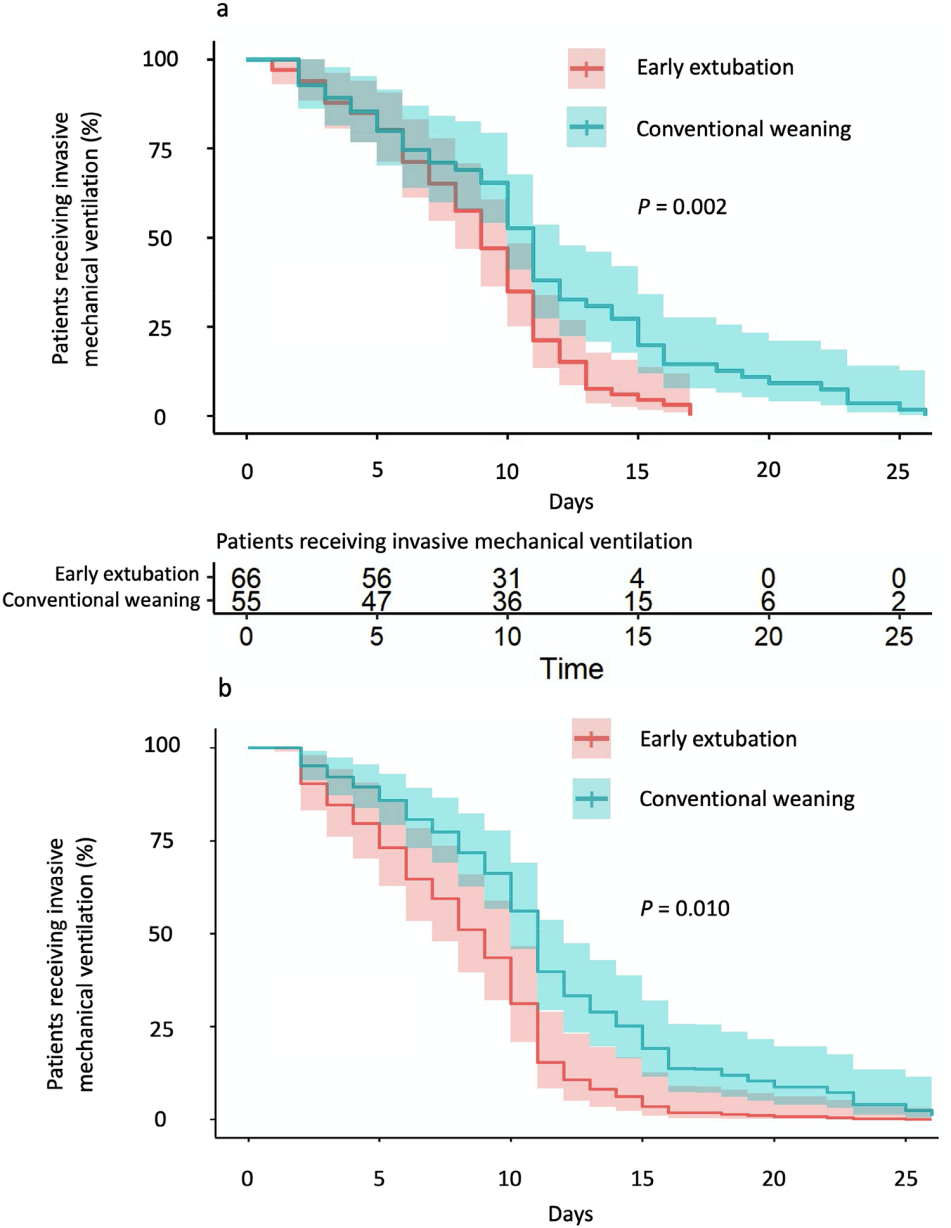
Kaplan–Meier curves indicating the time lag from intubation to IMV discontinuation. The Kaplan–Meier curve showing median and interquartile range are depicted: red line represents early extubation group while turquoise line refers to standard weaning group; *P *values refer to Log-Rank test. Kaplan–Meier curve constructed without (**a**) or with adjusting for propensity score (**b**).

The Kaplan–Meier curves indicating the time to liberation from IMV are depicted in Fig. 2.

The Kaplan–Meier curve showing median and interquartile range are depicted: red line represents early extubation group while turquoise line refers to standard weaning group; *P*-values refer to Log-Rank test. Kaplan–Meier curve constructed without (a) or with adjusting for propensity score (b).

According to Kaplan–Meier analysis (Fig. 2a), IMV was interrupted after 11.0 [6.0–15.0] days in the standard weaning group and after 9.0 [6.0–11.0] days in the early extubation group (*p* = 0.002). When the Kaplan–Meier curve was constructed adjusting for propensity score (Fig. 2b), IMV duration was of 11.0 [10.0–12.0] days in the standard weaning group and 9.0 [7.0–10.0] days in the early extubation group (*p* = 0.010).

The main medical treatments employed during ICU stay are listed in Table 3. In the standard weaning group, the use of anti-viral medications was less frequent (*p* = 0.004), while there was a higher use of steroids than in the early extubation group (*p* = 0.044), respectively. The mechanical ventilation settings and advanced therapy are reported in Supplementary material 1 (for missing data eTable1).

**Table 3:** Medical treatments.

	Standard weaning (n = 55)	Early extubation (n = 66)	*P-*value
Hydroxychloroquine, n (%)	49 (89.1)	62 (93.9)	0.509
Antiviral medications, n (%)	36 (65.5)	58 (87.9)	0.004
Antibiotics, n (%)	47 (85.5)	60 (90.9)	0.401
Steroids, n (%)	37 (67.3)	32 (48.5)	0.044
Anticoagulation, n (%)	37 (67.3)	37 (56.1)	0.262
Immunosuppressors, n (%)	7 (12.7)	9 (13.6)	> 0.999

Data are presented as number and percentage of patients (in brackets) *P* values refer to Fisher’s exact test.

In the standard weaning group, SBT duration was of 40.0 [30.0–60.0] min. SBT was conducted through T-tube or PSV + CPAP mode in more than 70% of the cases (eFigure 1). In the same group, IMV was discontinued after 1.0 [1.0–2.0] SBT attempts (range 1.0–3.0).

In the standard weaning group, prophylactic NIV was used in 33 (60.0%) patients (eTable2), while 16 (29.1%) patients received NIV as rescue treatment of post-extubation respiratory failure. When NIV was used in standard weaning group, its duration was of 2.0 [0.5–4.0] days, whereas, in patients early extubated, NIV duration was of 2.0 [1.0–4.3] days (*p* = 0.554). In the early extubation group, NIV was administrated soon after extubation through PSV in 52 (78.8%) patients and or CPAP mode in 14 (21.2%) patients.

Blood tests at hospital admission and during ICU stay are presented in Table 4 (for missing data eTable3). The two groups were homogenous for white cells and lymphocytes counts as well as for lactate-dehydrogenase and procalcitonin serum concentration and PaO_2_/FiO_2_. At extubation C-reactive protein serum levels were lower in the standard weaning group than in the early extubation group (*p* = 0.0003). In both groups PaO_2_/FiO_2_ at extubation was improved compared to hospital admission (*p* < 0.0001 for all comparisons) and time of intubation (*p* < 0.0001 for all comparisons).

**Table 4:** Blood tests at hospital admission and in course of intensive care unit stay.

	Hospital admission	Intubation	Extubation	*P* value
White cells count, × 10^3^/µL				
Standard weaning	7.95 [6.14–9.25]	7.70 [6.04–10.48]	11.01 [8.02–13.62]^†^,^‡^	< 0.0001
Early extubation	8.00 [6.00–11.38]	8.08 [5.57–11.69]	10.05 [6.89–14.00]^§^	
Lymphocytes count, × 10^3^/µL				
Standard weaning	0.74 [0.47–1.05]	0.70 [0.38–0.89]	1.13 [0.80–1.51]	0.669
Early extubation	0.93 [0.50–1.30]	0.58 [0.48–1.00]	0.86 [0.60–1.36]	
Reactive C-protein, mg/dL				
Standard weaning	15.03 [6.98–22.33]	17.50 [10.00–24.00]	3.19 [1.48–6.93]*,^†^,^‡^	0.0002
Early extubation	14.6 [6.74–18.13]	16.00 [9.50–21.00]	10.00 [5.10–17.00]^††^	
Lactate–dehydrogenase, U/L				
Standard weaning	646.50 [452.00–869.80]	600.00 [412.50–867.50]	473.00 [324.00–622.00]^†^,^‡‡^	< 0.0001
Early extubation	533.00 [358.00–903.50]	564.00 [389.5–780.50]	395.00 [271.00–530.00]^§§^,^†††^	
Procalcitonin, ng/mL				
Standard weaning	0.36 [0.15–1.16]	0.26 [0.12–0.63]	0.13 [0.07–0.23]	0.685
Early extubation	0.55 [0.20–1.60]	0.55 [0.18–1.53]	0.22 [0.10–0.50]	
PaO_2_/FiO_2_, mmHg				
Standard weaning	88.62 [67.25–113.20]	118.30 [88.75–149.00]^‡‡‡^	250.00 [187.80–319.30]^†^,^‡^	< 0.0001
Early extubation	111.20 [78.77–142.90]	137.50 [102.70–196.50]	213.30 [189.80–284.00]^†^,^‡^	

Data are presented as median and [interquartile range]. PaO_2_/FiO_2_, arterial oxygen tension to inspired oxygen fraction ratio. *P* values refer to weaning category-time interactions from Satterthwaite's analysis of variance whereas symbols refer to *P* values from post hoc Tukey method for comparison: *P = 0.0003 conventional weaning versus early extubation; ^†^*P* < 0.0001 extubation versus hospital admission; ^‡^*P* ≤ 0.0001 extubation versus intubation; ^§^*P* = 0.031 extubation versus intubation; ^††^*P* = 0.020, extubation versus intubation;

^‡‡^*P* = 0.0005, extubation versus intubation; ^§§^*P* = 0.012 extubation versus hospital admission; ^†††^*P* = 0.001, extubation versus intubation; ^‡‡‡^*P* = 0.029 intubation versus hospital admission.

## Discussion

In the present retrospective multicentric investigation in patients undergoing IMV for severe COVID-19 related ARF, early extubation followed by immediate NIV application improved clinical outcomes respect to standard weaning strategy with SBT, by reducing the length of IMV and decreasing both extubation failure and reintubation rate, without affecting ICU length of stay and ICU mortality. These results are of particular interest in the COVID-19 pandemic, characterized by an imbalance between the high number of patients needing immediate ventilatory assistance and the prompt availability of ICU beds and ventilator machines^12–14^. While reducing the risk of pulmonary infections is important for all intubated patients regardless of the underlying disease, reducing the days of mechanical ventilation and the length of ICU stay is of paramount importance when high numbers of patients require ICU admission for receiving IMV, determining the need for elevated ICU surge capacity, as it occurs during the COVID-19 outbreak^12–14^. Indeed, any strategy aimed to improve the prompt availability of ICU beds and ventilators is desired^12–14^.

NIV for facilitating IMV discontinuation has been suggested by guidelines only in patients admitted for hypercapnic ARF^9^ since it reduces the incidence of tracheostomy and ventilator-associated pneumonia, and shortens IMV duration, ICU, and hospital length of stay, compared to standard weaning^15^. Recently, however, in selected patients recovering from hypoxemic ARF, the application of NIV, immediately after early extubation, demonstrated to reduce the length of IMV, as opposed to standard weaning^10,11^. The present investigation suggests that COVID-19 patients may also benefit of this approach to weaning. Moreover, differently from the aforementioned study on hypoxemic ARF patients^10,11^, in our COVID-19 patients early extubation was able to significantly decrease the rate of extubation failure and reintubation, compared to standard weaning.

Extubation failure approximatively occurs in 10–15% of cases, in patients deemed ready for IMV discontinuation^16^ and in more than 20% of the cases, in patients considered at risk for reintubation^16,17^. NIV is recommended to prevent extubation failure in patients considered at risk for reintubation^9^, as it has been demonstrated that the prophylactic use of NIV is able to reduce the rate of reintubation, compared to standard oxygen therapy^18,19^. Also, when NIV is combined with high flow nasal oxygen therapy in patients older than 65 years or with an underlaying cardiac or respiratory disease, the rate of reintubation is lower than with high flow nasal oxygen therapy only^20^. Previous works^18–20^ have reported that the rate of reintubation with NIV ranged from 5 to 8.3% within the 48 h after extubation and achieved 12% at ICU discharge. In our population, the application of NIV immediately after early extubation decreased the rate of extubation failure and reintubation compared to standard weaning group, despite 60% of the patients were prophylactically assisted after IMV discontinuation. However, in our setting, failure of a planned extubation and need for reintubation occurred much more frequently than previously described in mixed populations of hypoxemic and hypercapnic ARF patients^18–20^. Indeed, the 40% reintubation rate is definitely high, compared to the usual rates reported in studies not involving COVID-19 patients^18–20^. That said, to our knowledge, no study has reported insofar the rate of extubation failure and reintubation in COVID-19 patients. In a recent RCT aimed at comparing standard weaning vs. early extubation + immediate NIV application in a population of non-COVID-19 hypoxemic patient recovering from non-hypercapnic acute respiratory failure^11^, we had a rate of treatment failure in the conventional weaning group of 18%, which is definitely lower than the 40% reported here. Noteworthy, the rate of failure in the early extubation + NIV group in that study was 8%^11^, which is also much lower than the 18.2% reported here. In our series, the vast majority of early extubation + NIV data were collected in centers also enrolling for the aforementioned study^11^, which indirectly suggests that the rate of extubation failure in COVID-19 patients might exceed that generally observed in non-COVID-19 patients.

This multicenter retrospective investigation has several limitations that must be considered. First, data were retrospectively obtained from medical records with a limited sample size. Accordingly, the lack of a randomization in study population sampling—i.e., sampling bias—could make our results not generalized to other settings. Second, the participating centers that applied a protocol of early extubation, were highly skilled in NIV assistance, which might raise questions about generalizability. Third, SBT was conducted in more than 45% of the cases in T-tube mode. This procedure might have adversely affected the weaning outcome as previously described^21^. Also, the progressive reduction of ventilatory assistance in standard weaning group could have negatively affected clinical outcomes. Fourth, also the rate of ventilator-associated pneumonia, tracheobronchitis, and other severe events, such as pneumothorax, pulmonary embolism, and hemorrhagic, septic, cardiac, renal, or neurologic episodes were not recorded. Fifth, knowing how resource availability has changed during the study period is an interesting piece of information that might have influenced physician’s attitude for IMV and NIV. Unfortunately, we did not record this information. Unfortunately, we did not record this information. Finally, our results should be considered specific for COVID-19 population, and thus are not generalizable under other conditions.

In conclusion, in scenarios needing too early extubation as that of our cohort of patients receiving IMV for severe COVID-19 related hypoxemic ARF, direct extubation to NIV seems to perform better than ordinary SBT because it has the potentiality to improve clinical outcomes compared to standard weaning, by shortening IMV duration and reducing the rate of extubation failure and reintubation.

## Methods

The study was approved by local Ethics Committees of coordinator center (Comitato Etico Interaziendale Novara, Italy – CE 120/20) and collaborators hospitals. The present multicenter retrospective study was conducted according to the Helsinki Declaration principles in 12 ICUs, 6 of which were highly skilled in early extubation followed by immediate NIV application. ﻿Due to the retrospective nature of the investigation, the need for informed consent from individual patients was waived by local Ethics Committees of coordinator center (Comitato Etico Interaziendale Novara, Italy – CE 120/20) and collaborators hospitals.

### Patients

All consecutive adult patients, intubated for severe COVID-19 related ARF from March 1^st^ to April 30^th^, 2020, and subsequently weaned from IMV, were considered eligible. ﻿Laboratory confirmation for SARS-CoV-2 infection was defined as a positive result of reverse transcriptase-polymerase chain reaction (RT-PCR) assay of naso-pharyngeal swabs obtained on hospital admission. In case of negative result, RT-PCR assay from lower respiratory tract aspirate/bronco-alveolar lavage was carried out after intubation in ICU^22^.

Patients were evaluated for IMV discontinuation when they met the following criteria: (1) assisted ventilation mode with a total inspiratory pressure < 30 cmH_2_O; (2) respiratory rate ≤ 30 breathes*min^-1^; (3) effective cough; (4) core temperature ≤ 38.5 °C; (5) arterial partial pressure of oxygen (PaO_2_) to inspired oxygen fraction (FiO_2_) ratio (PaO_2_/FiO_2_) ≥ 150 mmHg or peripheral oxygen saturation (SpO_2_) between 90 and 94% with FiO_2_ ≤ 0.6; (65) arterial pH ≥ 7.35; 6) Glasgow Coma Scale ≥ 11 (V1tube). Patients were excluded in case of IMV lasting < 24 h and contraindications to NIV^23^.

The study population was divided in two groups according to weaning plan adopted:

1. Standard weaning group, where extubation was performed only after having encompassed SBT in pressure support ventilation (PSV), neurally adjusted ventilatory assist (NAVA), continuous positive end-expiratory pressure (CPAP), or T-tube mode^7^.
2. Early extubation group, where no SBT was performed and patients were early extubated and immediately supported through facial mask or helmet NIV either in PSV or CPAP mode^10,11^.

In the standard weaning group, patients underwent a progressive reduction of positive end-expiratory pressure (PEEP) and inspiratory pressure support during assisted mode. When PEEP ≤ 10 cmH_2_O and inspiratory pressure support over PEEP ≤ 12 cmH_2_O or NAVA gain ≤ 0.8 cmH_2_O*µV^-1^, a 30–60-min-lasting SBT was performed. Once SBT was passed according to each center institutional weaning protocol criteria, detailed in the online Supplementary material 2, patients were extubated and allowed to spontaneously breath through Venturi mask or high flow nasal cannula, with additional oxygen to maintain SpO_2_ between 90 and 94%. After extubation, NIV was used either in CPAP or PSV (1) as prophylactic ventilatory assistance in patients judged at high risk for extubation failure^9,24^, and (2) as a rescue therapy in case of post-extubation respiratory failure.

Conversely, in case of SBT failure, the assisted ventilatory mode was restored and a new SBT was attempted after 24 h.

In early extubation group, when PEEP and inspiratory pressure support over PEEP were both ≤ 15 cmH_2_O with a total inspiratory pressure < 30 cmH_2_O during assisted ventilatory mode, in presence of a respiratory rate ≤ 30 breathes*min-1, an effective cough, a core temperature ≤ 38.5 °C, a PaO2/FiO2) ≥ 150 mmHg or SpO2 between 90 and 94% with FiO2 ≤ 0.6, an arterial pH ≥ 7.35, and Glasgow Coma Scale ≥ 11 (V1tube), patients were extubated without SBT and NIV was immediately started either in PSV or CPAP mode. During NIV, the values of PEEP and inspiratory pressure support were set according to those previously set during IMV, while FiO_2_ was chosen to assure SpO_2_ varying from 90 to 94%. NIV pressure support and PEEP were progressively reduced and spontaneous breathing was allowed, with oxygen supplementation as previously described.

In both groups extubation failure was defined as the need for reintubation within the 48 h after extubation^25^. In the conventionally weaning group additional extubation failure criteria were considered the needing of NIV within 48 h after extubation. Prophylactic NIV and high flow nasal cannula were not considered extubation failure^11^.

The centers involved in the study adhered to one weaning strategy or the other according to their internal protocols.

### Data collection

Clinical data were retrospectively acquired in the centers that adhered to the present investigation and sent to the coordinator center for analysis. The recorded data included the followings: age, sex, body mass index, medical comorbidities, gravity index at ICU admission (Simplified Acute Physiology Score II and/or Sequential Organ Failure Assessment) and anti-viral therapies. Length of IMV, extubation failure, reintubation and tracheostomy rate, as well as ICU length of stay and in-ICU mortality were computed. Both pre-intubation and post-extubation NIV duration were also noted. Mechanical ventilation settings (i.e., PEEP and plateau pressure after intubation, level of assistance before SBT or before early extubation) were obtained. PaO_2_/FiO_2_, total white blood cells, and lymphocytes counts as well as lactate-dehydrogenase, procalcitonin, and C-reactive protein serum concentration were recorded at hospital admission and on the day of intubation and extubation. Finally, the rate of prone positioning, nitrous oxide inhalation, and extra-corporeal membrane oxygenation application were acquired.

### Statistical analysis

Because of the retrospective nature of the study, no statistical sample size calculation was computed a priori. The study population was divided in two groups: 1) standard weaning with SBT and 2) early extubation followed by immediate NIV. Continuous variables were presented as median and interquartile range, whereas categorical variables were expressed as number and percentage. Comparison between groups was assessed using Mann–Whitney’s test for continuous variables and Fisher’s exact test for categorical variables. In case of missing data, the comparison was made only with available data.

Kaplan–Meier curves, depicting the time lag from intubation to IMV discontinuation in each group, were determined and compared trough Log-Rank test. A propensity score was estimated to balance the patient’s characteristics among groups. The covariate balancing propensity score estimation was computed considering baseline characteristics: age, gender, NIV before intubation, pharmacological treatments, body mass index, SAPSII, SOFA, comorbidities, smoke, and days from hospitalization to symptoms onset. The Cox Regression model for inverse probability weight propensity adjusted survival curve was also computed.

A generalized linear mixed model adjusted for inverse probability weight propensity score was computed evaluating the marginal and interaction effect of the time (hospital admission, intubation, extubation, and NIV) and weaning plan adopted on the haemato-chemical parameters; a random intercept term on the center and patient identification number was introduced in the model accounting for both correlation within repeated measurements and within the same center. For all comparisons, a p-value < 0.05 was considered significant. Statistical analysis was carried out using R 3.6.2 (The R Foundation).

### Ethics approval and consent to participate

The present investigation was approved by all the ethics committees of the centers that adhered to the study.

## Supplementary Information

Supplementary Information 1.

Supplementary Information 2.

## Data Availability

In accordance to ICMJE, the data sharing has been planned as follows: Whether individual, de-identified participant data (including data dictionaries) will be shared: Yes; What data in particular will be shared: Individual participant data that underlie the results reported in this article, after deidentification (text, tables and figures). Whether additional, related documents will be made available: No; When and for how long the data will become/be available: Beginning 9 months and ending 36 months following article publication. The criteria to access the data (including who can request access and for what types of analyses, and the name of the data repository): Researchers who provide a methodologically sound proposal for individual participant data meta-analysis should contact principal investigator to gmcamma@gmail.com. Proposals may be submitted up to 36 months following article publication.
