# Supplementary material for: Early extubation with immediate non-invasive ventilation versus standard weaning in intubated patients for coronavirus disease 2019: a retrospective multicenter study
Source: Sci Rep. 2021 Jun 28;11:13418. doi: 10.1038/s41598-021-92960-z (PMC8239031; doi:10.1038/s41598-021-92960-z)
Supplement: Supplementary file 1 — Supplementary Information 1. [file 41598_2021_92960_MOESM1_ESM.docx]

**Additional Files**

Additional material

.docx

Weaning criteria, Ventilator settings and advanced therapies, Spontaneous breathing trial modes, Missing data for ventilator settings, Indications for prophylactic non-invasive ventilation in standard weaning group, Missing data for blood tests at hospital admission and in course of intensive care unit stay, Propensity score and C-statistic, Common Support Propensity score plot, and Mean Difference measure in propensity adjusted and unadjusted results.
