# Supplementary material for: Early extubation with immediate non-invasive ventilation versus standard weaning in intubated patients for coronavirus disease 2019: a retrospective multicenter study
Source: Sci Rep. 2021 Jun 28;11:13418. doi: 10.1038/s41598-021-92960-z (PMC8239031; doi:10.1038/s41598-021-92960-z)
Supplement: Supplementary file 2 — Supplementary Information 2. [file 41598_2021_92960_MOESM2_ESM.docx]

**Title**

Early extubation with immediate non-invasive ventilation versus standard weaning in intubated patients for coronavirus disease 2019: a retrospective multicenter study.

**List of the Authors**

Gianmaria Cammarota, Rosanna Vaschetto MD, Danila Azzolina, Nello De Vita, Carlo Olivieri, Chiara Ronco, Federico Longhini, Andrea Bruni, Davide Colombo MD, Claudio Pissaia, Federico Prato, Carlo Maestrone, Matteo Maestrone, Luigi Vetrugno, Tiziana Bove, Francesco Lemut, Elisa Taretto, Alessandro Locatelli, Nicoletta Barzaghi, Martina Cerrano, Enrico Ravera, Christian Zanza, Andrea Della Selva, Ilaria Blangetti, Francesco Salvo, Fabrizio Racca, Yaroslava Longhitano, Annalisa Boscolo, Ilaria Sguazzotti, Valeria Bonato, Francesca Grossi, Federico Crimaldi, Raffaella Perucca, Ester Boniolo, Federico Verdina, Gian Luca Vignazia, Erminio Santangelo, Riccardo Tarquini, Marta Zanoni, Antonio Messina, Matteo Marin, Paola Bacigalupo MD^10^, Graziana Sileci, Nicolò Sella, Edoardo De Robertis, Francesco Della Corte, Paolo Navalesi.

Additional Material

**Supplementary material 1.** Ventilator settings and advanced therapies.

Immediately after intubation, plateau airway pressure was 22.0 [20.0–24.0] cmH_2_O in the standard weaning group versus 24.0 [22.0–25.3] cmH_2_O in the early extubation group (p=0.011).

At intubation, PEEP was similar in the two groups (standard weaning 12.0 [10.0–15.0] cmH_2_O versus early extubation 14.0 [12.0–15.0] cmH_2_O, p = 0.225) whereas at extubation, PEEP was lower in standard weaning group than in early extubation group (6.0 [5.0–8.0] cmH_2_O versus 10.0 [8.0–11.8] cmH_2_O, p<0.0001). Only in standard weaning group, PEEP decreased moving from intubation to extubation (p<0.0001).

At extubation, pressure support was lower in standard weaning group versus early extubation group (8.0 [6.0–10.0] cmH_2_O versus 10.0 [7.0–10.0] cmH_2_O, p = 0.041), while FiO_2_ was similar among groups (0.40 [0.32–0.45] in conventional weaning group versus 0.40 [0.35–0.50] in early extubation group, p = 0.966). Prone positioning [39 (70.9%) versus 48 (72.7%) cases (p = 0.842)] and nitrous oxide inhalation [5 (9.1%) versus 1 (1.5%) cases (p = 0.091)] were equally applied in both groups. No patients required extra-corporeal membrane oxygenation therapy.

Supplementary material 2. Weaning criteria part 1of 2.

| **Failure of Spontaneous breathing trial** |
| --- |
|  |
| 1. Agitation and anxiety. |
| 1. Diaphoresis, cyanosis, evidence of increased breathing effort (activation of accessory respiratory muscles), dyspnea. 2. Abundant secretions; |
| 1. Systolic blood pressure >190 mm Hg; Mean arterial pressure <60 mmHg; heart rate >140 beats*min^-1.^ |
| 1. PaO_2_/FiO_2_﻿ ﻿≤ 150 mm Hg or arterial oxygen saturation <90% with FiO_2_ ≥0.6. |
|  |
| 1. Respiratory rate ≥35 breathes*min^-1^; Respiratory rate on tidal volume ratio >105 breathes*min^-1^*l^-1^. |
| 1. pH <7.32 or a decrease in pH ≥0.07 units. |
| 1. Cardiac arrhythmias or electrocardiographic signs of ischemia. |

PaO_2_/FiO_2_, arterial oxygen tension on inspired oxygen fraction ratio; FiO_2_, inspired oxygen fraction; PaCO_2_, arterial carbon dioxide tension.

| **Supplementary material 2.** Weaning criteria part 2 of 2 | |
| --- | --- |
| **Post-extubation failure criteria** | |
|  | |
| Respiratory rate >25 breathes*min^-1^ over at least 2 hours. | |
| Heart rate >140 beats*min^-1^ or sustained increase or decrease >20%. | |
| Clinical signs of respiratory muscle fatigue (activation of accessory respiratory muscles). | |
| SaO_2_ <90%; PaO_2_/FiO_2_ <250 with FiO_2_ ≥0.5. | |
| PaCO_2_ >45 mm Hg or ≥ 20% from pre-extubation; pH <7.35. | |
|  | |
| **Criteria for non-invasive ventilation** | |
|  | |
| Non-invasive CPAP: dyspnea and PaO_2_/FiO_2_ <200 mm Hg. | |
| Non-invasive ventilation: ﻿dyspnea with 7.30 >pH <7.35 and 45 mm Hg <PaCO_2_ <50 mm Hg. | |
|  | |
| **Criteria for reintubation** | |
|  |  |
| Cardiac or respiratory arrest. | |
| Inability to protect airway; coma or psychomotor agitation not controlled by continuous intravenous sedative infusion. | |
| Unmanageable secretions or uncontrolled vomiting. | |
| Life threatening arrhythmias or electrocardiographic signs of ischemia. | |
| Hemodynamic instability (mean arterial pressure <60 mm Hg despite fluids and or low dosage vasopressors administration). | |
| Intolerance to all interfaces for non-invasive ventilation. | |
| Two of the following: dyspnea, PaO_2_/FiO_2_ <200 mm Hg, and respiratory acidosis (pH <7.30 and PaCO_2_ >50 mm Hg). | |

SaO_2_, arterial oxygen saturation, PaO_2_/FiO_2_, arterial oxygen tension on inspired oxygen fraction ratio; FiO_2_, inspired oxygen fraction; PEEP, positive end-expiratory pressure; PaCO_2_, arterial carbon dioxide tension; CPAP, continuous positive end-expiratory pressure.

**eFigure 1**. Spontaneous breathing trial modes


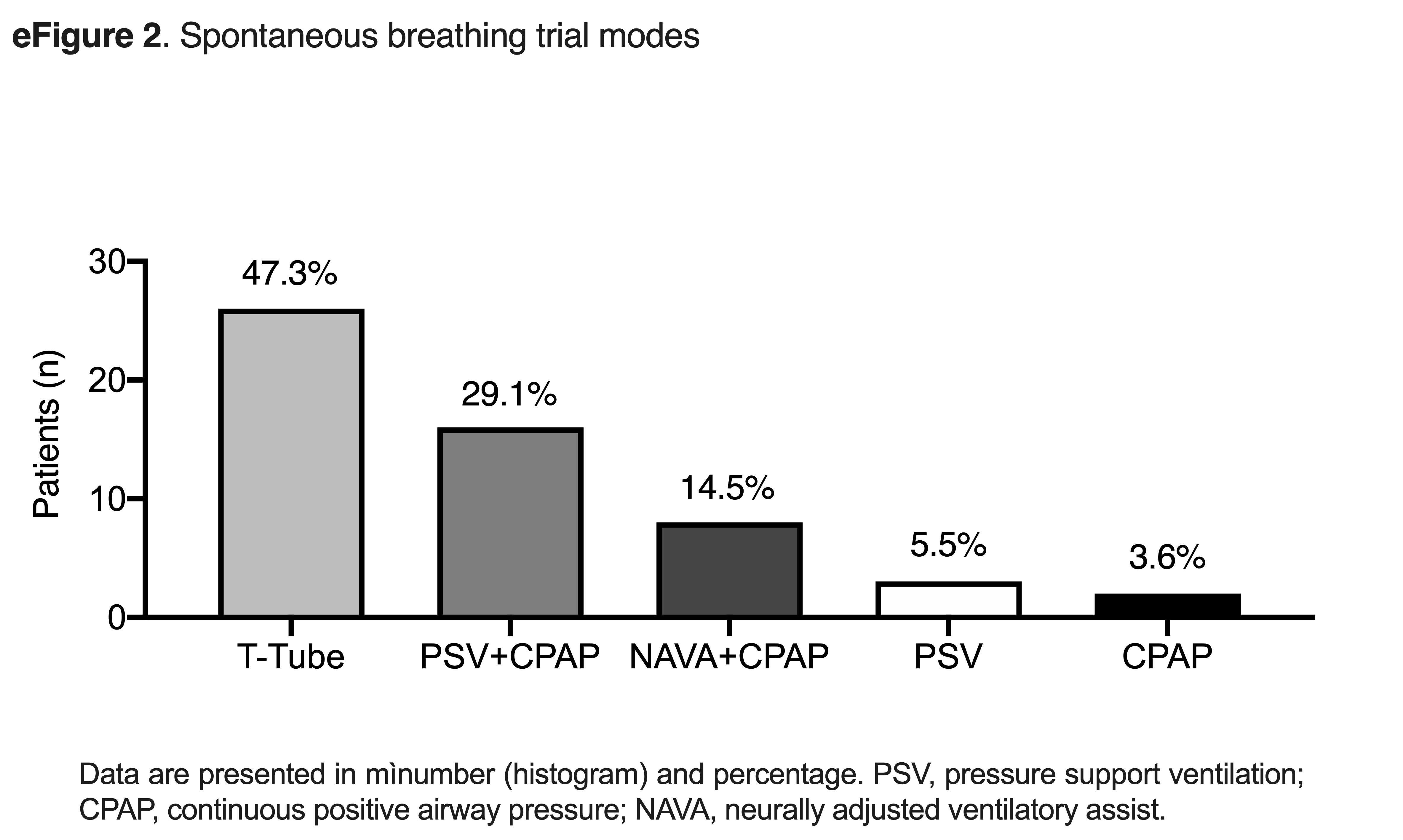


Data are presented in number and percentage of patients. PSV, pressure support ventilation; CPAP, continuous positive airway pressure; NAVA, neurally adjusted ventilatory assist.

**eTable 1.** Missing data for ventilator settings

|  | **Intubation** | **Extubation** |
| --- | --- | --- |
| PEEP |  |  |
| Standard weaning | 0 | 2 |
| Early extubation | 1 | 2 |
| Plateau of airway pressure |  |  |
| Standard weaning | 4 | - |
| Early extubation | 20 | - |
| Pressure support |  |  |
| Standard weaning | - | 5 |
| Early extubation | - | 7 |
| Inspiratory oxygen fraction |  |  |
| Standard weaning | - | 1 |
| Early extubation | - | 2 |

Missing data are reported for each variable in the two groups across all the study phases. PEEP, positive end-expiratory pressure.

**eTable 2.** Indications for prophylactic non-invasive ventilation in standard weaning group

|  | **Standard weaning**  **(n = 55)** |
| --- | --- |
| Cardiac disease, n (%) | 6 (10.9) |
| Chronic obstructive pulmonary disease, n (%) | 3 (5.5) |
| Age > 65 years, n (%) | 12 (21.8) |
| Mechanical ventilation > 7 days, n (%) | 7 (12.7) |
| Clinical judgment, n (%) | 5 (9.1) |

Data are presented as number and percentage (brackets).

**eTable 3.** Missing data for blood tests at hospital admission and in course of intensive care unit stay

|  | **Hospital admission** | **Intubation** | **Extubation** |
| --- | --- | --- | --- |
| White cells count, x10^3^/µL |  |  |  |
| Standard weaning | 5 | 7 | 12 |
| Early extubation | 11 | 1 | 3 |
| Lymphocytes count, x10^3^/µL |  |  |  |
| Standard weaning | 6 | 7 | 18 |
| Early extubation | 9 | 1 | 3 |
| Reactive C-protein, mg/dL |  |  |  |
| Standard weaning | 5 | 7 | 13 |
| Early extubation | 5 | 3 | 3 |
| Lactate–dehydrogenase, U/L |  |  |  |
| Standard weaning | 7 | 8 | 14 |
| Early extubation | 26 | 10 | 13 |
| Procalcitonin, ng/mL |  |  |  |
| Standard weaning | 8 | 12 | 15 |
| Early extubation | 36 | 10 | 16 |
| PaO_2_/FiO_2_, mmHg |  |  |  |
| Standard weaning | 6 | 8 | 14 |
| Early extubation | 15 | 0 | 3 |

Missing data are reported for each variable in the two groups across all the study phases. PaO_2_/FiO_2_, arterial oxygen tension to inspired oxygen fraction ratio.

**Supplementary material 3.** Propensity score and C-statistic

Once a propensity score has been calculated a Common Support Balance plot has been reported to ensure that there is overlap in the range of propensity scores across intervention groups. In a propensity score analysis, the treatment effects could be properly assessed for a treated individual for whom there is not a comparison individual with a similar propensity score.

The C-statistic measure has been also computed for the imbalance assessment. In the absence of baseline imbalance, the propensity score has a normal distribution of mean 0.5 in each group of the study, and thus the baseline variables are independent from the intervention allocation. In other words, the c-statistic of the propensity score model is close to 0.5. By contrast, if at least one covariate is associated with intervention allocation, the c-statistic will be larger than 0.5. The C-Statistic for linear regression model predicting the treatment allocation according to patients’ characteristics has been computed before and after the propensity score weighting adjustment ^1^.

Mean differences diagnostics have been also reported because they were the most commonly used propensity score diagnostic. Standardised differences are preferred because they are independent of sample size and are a property of the sample ^2^. It has been suggested that a standardized difference of less than 0.1 can be considered as adequate balance ^3^.

**References**

1. Leyrat C, Caille A, Foucher Y, Giraudeau B. Propensity score to detect baseline imbalance in cluster randomized trials: the role of the c-statistic. BMC Med Res Methodol. 2016;16(1):9. doi:10.1186/s12874-015-0100-4

2. Granger E, Watkins T, Sergeant JC, Lunt M. A review of the use of propensity score diagnostics in papers published in high-ranking medical journals. BMC Med Res Methodol. 2020;20(1):132. doi:10.1186/s12874-020-00994-0

3. Normand S-LT, Landrum MB, Guadagnoli E, et al. Validating recommendations for coronary angiography following acute myocardial infarction in the elderly. Journal of Clinical Epidemiology. 2001;54(4):387-398. doi:10.1016/S0895-4356(00)00321-8

**eFigure 2.** Common Support Propensity score plot.


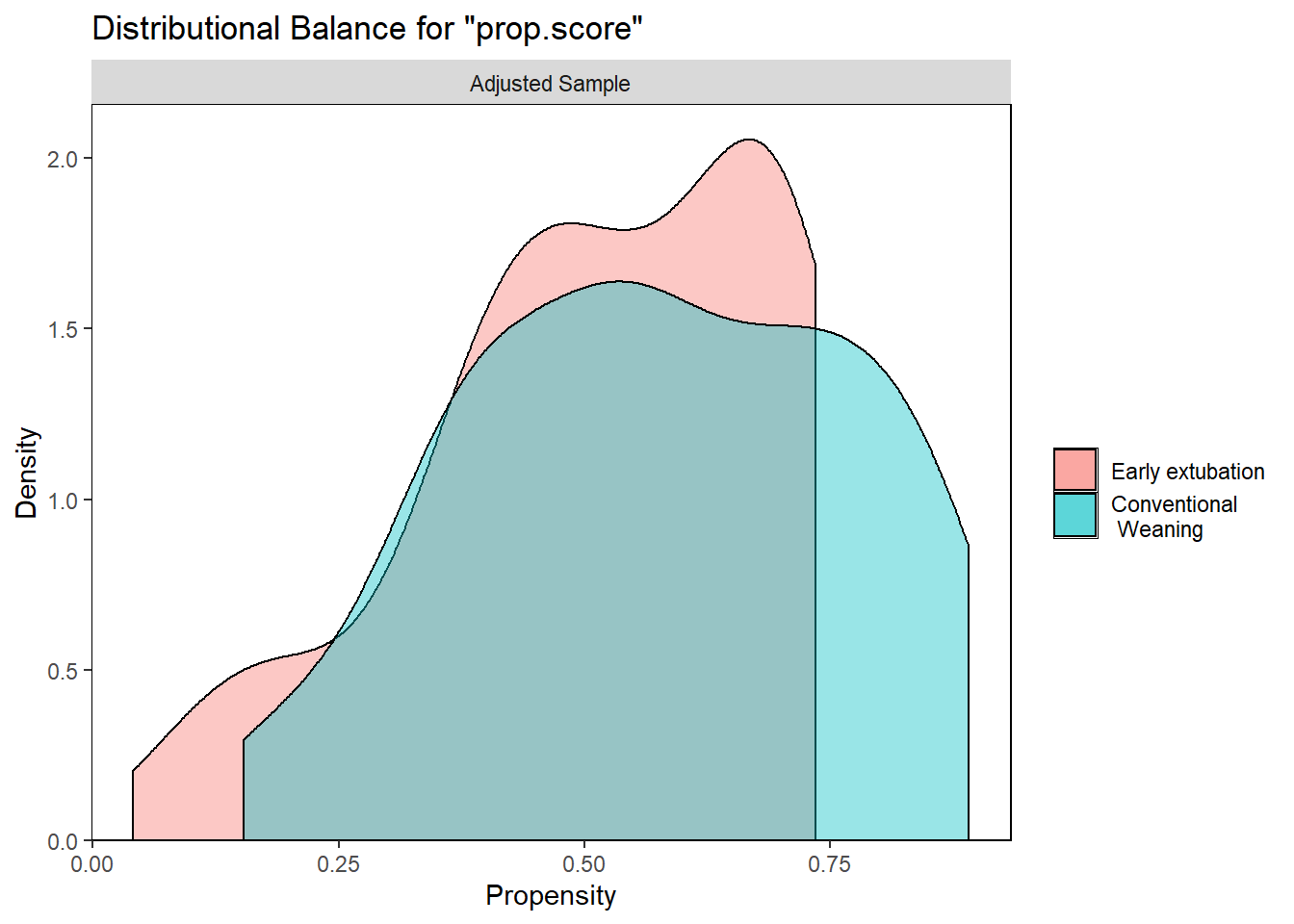


Density estimates of the propensity score values have been reported according to Early extubation and Conventional weaning groups. The overlap of the propensity scores distributions across groups is displayed in the figure. We found the extent of overlap to be satisfactory.

The propensity balance assessment has been also performed by considering the C-statistics measure. The results in the C-statistic of the model predicting assignment to the intervention without propensity adjustment is 0.90 indicating a baseline imbalance in patient characteristics. Indeed, the C-statistics for the propensity adjusted model is instead 0.56 (c-statistics close to 0.5 indicate successful balance) indicating that the propensity score could adequately handle the imbalances between treatment groups assignment.

**eFigure 3.** Mean Difference measure in propensity adjusted and unadjusted results
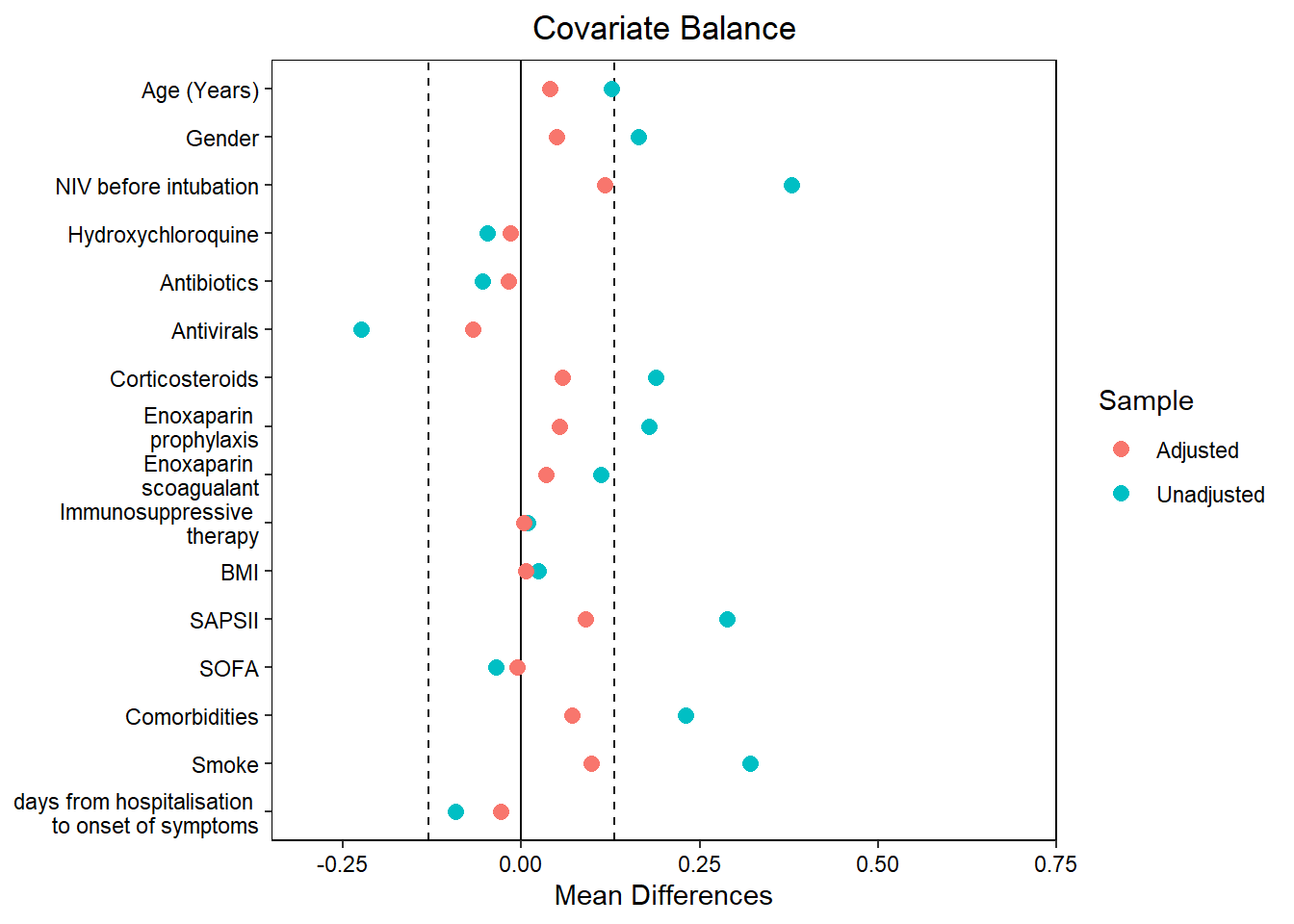


The differences between the propensity-adjusted means are generally within the limit of 0.1 indicating a good balance of the propensity score concerning the covariates. BMI, body mass index, SAPSII, simplified acute physiology score II; SOFA, sequential organ failure assessment; NIV, non-invasive ventilation.
